# Supplementary material for: Human Copper-Containing Amine Oxidases in Drug Design and Development
Source: Molecules. 2020 Mar 12;25(6):1293. doi: 10.3390/molecules25061293 (PMC7144023; doi:10.3390/molecules25061293)
Supplement: Supplementary file 1 [file molecules-25-01293-s001.pdf]

Table S1. List of traditional and IUPAC names of the compounds referred to in the text

Compound	Name from the original study	IUPAC name
ASP8232	—	— (Undisclosed Structure)
BTT-2027	—	Trans-2-(1-methyl-hydrazino)-1-indanol
BTT2052	—	(1S,2S)-2-(1-methyl-hydrazino)-indanol (dibasic)
Hydralazine	—	1-hydrazinylphthalazine
LJP1207	—	N-(2-phenylallyl)hydrazine hydrochloride
LJP1586	—	Z-3-fluoro-2-(4-methoxybenzyl)allylamine; 6 3-(3,4-diphenyl-1,3-oxazol-2-yl)propanal oxime
PRX167700	—	— (Undisclosed Structure)
PXS-4681A	—	4-[[(2E)-4-Amino-2-fluoro-2-buten-1-yl]oxy]-N-cyclohexyl benzamide
PXS 4728A (BI 1467335)	—	4-[(E)-2-(aminomethyl)-3-fluoroprop-2-enoxy]-N-tert-butylbenzamide
SzV-1287	—	3-(3,4-diphenyl-1,3-oxazol-2-yl)propanal oxime
TERN-201	—	— (Undisclosed Structure)
R1	2a,b	2-hydrazinyl-1-phenylethan-1-ol
R2	8	2-hydrazinyl-1-phenylpentan-1-ol
R3	12	2-(1-methylhydrazin-1-yl)-1-phenylpentan-1-ol
R4	11a-d	2-(1-methylhydrazin-1-yl)-1,2-diphenylethan-1-ol
R5	10	N-{4-[2-(4-carbamimidamidophenyl)ethyl]-1,3-thiazol-2-yl}acetamide
R6	35a	N-{4-[2-(4-carbamimidamidophenyl)ethyl]-5-(4-methanesulfonylphenyl)-1,3-thiazol-2-yl}acetamide
R7 (U-V002)	35c	N-{4-[2-(4-carbamimidamidophenyl)ethyl]-5-[(4-methanesulfonylphenyl)methyl]-1,3-thiazol-2-yl}acetamide
R8	19	4-benzyl-1H-imidazol-2-amine
R9	37b	N-[4-(2-{3-[(2-amino-1H-imidazol-4-yl)methyl]phenyl}ethyl)-1,3-thiazol-2-yl]acetamide
R10 (PXS-4159A)	28	4-{[(2E)-4-amino-2-fluorobut-2-en-1-yl]oxy}-N-Cyclohexylbenzamide
R11	ELP12	[(2-methoxyphenyl)methyl][6-(4-{2-[1-(6-{[(2-methoxyphenyl)methyl]amino}hexyl)piperidin-4-yl]ethyl}piperidin-1-yl)hexyl]amine
R12	4a	2-amino-N-({[1,1'-biphenyl]-3-yl}methyl)-N-methylacetamide
R13	4g	2-amino-N-methyl-N-{[4'-(morpholin-4-yl)-[1,1'-biphenyl]-3-yl]methyl}acetamide
R14	17h	4-[4-(5-{3-[(2-amino-N-methylacetamido)methyl]phenyl}pyrimidin-2-yl)piperazin-1-yl]-3-chlorobenzoic acid
R15	6	5-(cyclohexylamino)-2-phenyl-6-(1H-1,2,4-triazol-3-yl)-2,3-dihydropyridazin-3-one
R16	7	2-phenyl-5-[(propan-2-yl)amino]-6-(1H-1,2,4-triazol-3-yl)-2,3-dihydropyridazin-3-one
R17	13	2-(4-chlorophenyl)-5-{[4-(4-methylpiperazin-1-yl)phenyl]amino}-6-(1H-1,2,4-triazol-3-yl)-2,3-dihydropyridazin-3-one

Remark: all preferred IUPAC names were generated using "Structure to Name" tool in Chemaxon MarvinSketch 19.9.

Table 2. List of amino acids relevant to inhibitor binding that differ among VAP-1 orthologs and copper-containing amine oxidase sub-families.

Human VAP-1 residues	Rat VAP-1 residues	Murine VAP-1 residues	Human AOC2 residues	Human AOC1 residues	Inhibitors and interactions with the residue
Phe173	Thr	Asp	Arg	Thr	R10(hf), R11(hf), R17(pp)
Asp180	Gln	Glu	Trp	Tyr	R9(wh), R11(sb), R16(hb)
Thr210	Lys	Thr	Ala	Phe	R5(so), R9(so), R15(pp), R16(wh)
Thr212	Thr	Thr	Ala	Asp	R9(ph)
Phe389	Phe	Phe	Phe	Trp	R1(hf), R2(hf), R3(hf), R4(hf), R16(pp), BTT2052(hf)
Tyr394	Tyr	Tyr	Asn	Val	R1(hf), R2(hf), R3(hf), R10(hf), R11(hb+hf), R14(wh), R15(hb), R16(hb), R17(hb+pp)
Pro397	Pro	Pro	Gly	Glu	R11(hf)
Ile425	Leu	Leu	Leu	Tyr	R11(hf)
Asp446	Asp	Asp	Tyr	Ser	R7(hb), R11(sb), R14(ho)
Leu447	Phe	Phe	Leu	Asn	R6(ph), R7(hb), R9(ph), R11(hf), R12(hf), R12(ph), R14(hf), R15(pa), R16(pa), R17(pa)
Leu468	Leu	Leu	Val	Val	R1(hf), R4(hf), R12(wh), R13(wh), R14(wh), R17(pa)
Leu469	Leu	Leu	Gly	Tyr	R1(hf), R8(ph), R14(hf), R15(ps), R16(pa), R17(pa), BTT2052(hf)

Remark: The residues are colored by hydrophobicity (blue = most hydrophobic and red = most hydrophilic)

Interactions: hf – hydrophobic, hb – hydrogen bond, ho – halogen-oxygen interaction, wh – weak hydrogen bond, ph – π-proton interaction, pp – π-π-interactions, pa – π-alkyl interaction, ps – π-σ-interactions, sb – salt bridge, so – sulfur-oxygen interaction.
